# Real-world observational study on the long-term effect of L-glutamine treatment on renal parameters of adult and pediatric patients with sickle cell disease

**DOI:** 10.3389/fmed.2023.1243870

**Published:** 2023-12-06

**Authors:** Narcisse Elenga, Mohamed A. Yassin

**Affiliations:** ^1^Paediatric Department, Centre Hospitalier de Cayenne, Cayenne, France; ^2^Hematology Section, Medical Oncology Department, Hamad Medical Corporation, Doha, Qatar

**Keywords:** L-glutamine, sickle cell disease, clinical outcomes, hemolysis parameters, renal parameters

## Abstract

**Background:**

Sickle cell disease (SCD) is a rare genetic blood condition affecting millions worldwide. Oxidative stress is a key player in the pathogenesis of SCD and its comorbid consequences. Renal function impairment is a common complication of SCD in both pediatric and adult patients with serious consequences leading to increased risk of mortality. In this observational real-world study, we are reporting the long-term (120 weeks) renal function in 10 patients treated with L-glutamine.

**Methods:**

Ten patients (4 pediatric and 6 adults), with confirmed diagnoses of SCD (HbSS genotype), were enrolled, these included four patients from Qatar with Arab Indian haplotype and six patients from French Guiana with African haplotype. All patients were treated with L-glutamine oral powder (~0.3 g/kg body weight, Endari^®^) twice daily for 120 weeks. Clinical events and laboratory parameters (renal function, hemoglobin, reticulocytes, and lactate dehydrogenase [LDH]) were measured at baseline, 48, and 120 weeks.

**Results:**

The study showed that with L-glutamine treatment there were improvements in renal and hematological parameters with no vaso-occlusive crisis at both 48-and 120-week follow-up time points in all 10 patients. Improvements were seen in the albumin creatinine ratio (ACR) from baseline to 48 weeks (mean [Standard deviation SD] ACR: −4.19 [9.81] mg/g) and 120 weeks (mean [SD] ACR: −12.31 [21.09] mg/g). Mean (SD) increase in hemoglobin concentrations from baseline to 48 weeks and 120 weeks was 0.72 (1) g/dL and 1.41 (0.79) g/dL, respectively. Mean (SD) reticulocyte counts and LDH levels decreased from baseline to 48 weeks (mean [SD] change from baseline to 48 weeks, reticulocyte counts: −40.30 [101.58] × 10^9^ cells/L; LDH levels: −259 [154.93] U/L) and 120 weeks (mean [SD] change from baseline to 120 weeks, reticulocyte counts: −58.30 [128.38] × 10^9^ cells/L; LDH levels: −344.80 [274.63] U/L).

**Conclusion:**

This is one of the first studies that assessed the long-term renal outcomes in SCD using L-glutamine. L-glutamine improved the renal function in patients with SCD along with improvements in clinical outcomes and hemolysis, from 48 weeks and sustained through 120 weeks of treatment.

## Introduction

Sickle cell disease (SCD) is a rare genetic disorder affecting millions of people worldwide. SCD is caused by a single-point mutation of the β-globin gene (1). End-organ ischemia-reperfusion injury and infarction due to vaso-occlusion are the prominent pathophysiology of SCD that results in painful vaso-occlusive crisis (VOC) (2).

Hemodynamic alterations are correlated with chronic anemia and renal hypoxia due to vaso-occlusion and endothelial dysfunction in SCD. This results in the kidneys undergoing structural and functional changes (3). Renal function impairment is one of the most common complications in both adult and pediatric patients with SCD, which may lead to chronic kidney disease and in extreme cases progress to end-stage kidney disease (4). Sickle cell nephropathy (SCN) is a progressive disease that starts with glomerular hyperfiltration resulting in albuminuria, renal function impairment, and finally renal failure. In adult patients with SCD, renal failure due to SCN is one of the most frequent causes of mortality (4). Therefore, prompt diagnosis of SCN at an early age is crucial to facilitate timely treatment, thereby preventing renal impairment and subsequent renal failure.

The US FDA approved L-glutamine for treating SCD in patients aged 5 years and above in 2017. Earlier studies have demonstrated that long-term L-glutamine treatment (up to 120 weeks) significantly decreased SCD-related acute complications like a vaso-occlusive crisis (VOC) in patients with SCD (5, 6). It has been demonstrated that glutamine administration prevents apoptosis of immortalized human kidney 2 cells by inducing heme oxygenase-1 through a p38 mitogen-activated protein kinase (MAPK)-dependent mechanism, which could be crucial to the cytoprotective action of glutamine (7). Additionally, the primary glutamine consumers are immune cells and renal tubular cells (7). In this observational real-world study, we aimed to explore the long-term effect of L-glutamine on renal function in patients with SCD.

## Materials and methods

### Study design

This observational real-world study was conducted from October 2019 to April 2022 and included patients with confirmed SCD diagnoses enrolled under the Early Access Programs (EAPs). The local ethics committee (MRC- 04-20-1240 in Qatar and Commission Nationale Informatique et Libertés approval Number 3Yj157849 3 in French Guiana) approved the study. Participating patients or the parents or legal guardians of pediatric patients provided written informed consent. The primary study and long-term efficacy study are already published (5, 6). In brief, patients with confirmed SCD were treated with L-glutamine (~0.3 g/kg body weight per dose, Endari^®^, Emmaus Medical, Inc.) administered orally twice daily for 120 weeks. Clinical events and laboratory parameters [albuminuria, creatinine, LDH, hemoglobin, reticulocytes, estimated glomerular filtration rate (eGFR), and white blood cell (WBC)] were collected and measured at baseline, 48 and 120 weeks. VOCs were defined as acute severe pain that required hospitalization. The number of VOC (annualized rate of VOC) that occurred 12 months prior to starting treatment with L-glutamine was recorded as the baseline; at 120 weeks, the number of VOC was annualized to make it comparable to the number of VOC at baseline and 48 weeks Follow-up time points for both laboratory and clinical parameters were 48 and 120 weeks.

### Safety outcomes

Any adverse events (AEs) or serious adverse events (SAEs) that occurred while the patients were treated with Endari^®^ and subsequent clinic visits reported by patients and/or healthcare professionals were recorded.

### Statistical analysis

Statistical analysis was performed using XLSTAT Version 2021.3.1 (Lumivero). A paired *t*-test with *p* < 0.05 for statistical significance was performed by comparing the baseline data to 48-week and 120-week data.

## Results

Ten patients with confirmed SCD diagnosis (HbSS genotype) received treatment with L-glutamine oral powder (~0.3 g/kg body weight per dose, Endari^®^) twice daily for 120 weeks. Four patients were from Qatar with the Arab Indian haplotype, and six patients were from French Guiana with the African haplotype.

### Baseline characteristics

Of the total 10 patients with confirmed SCD diagnosis were included in this study, four (all adults) were from Qatar with Arab Indian haplotype, whereas six (four pediatric and two adults) were from French Guiana with African haplotype. The median age of patients was 22 years (range 10–37 years) with four patients below 18 years and six patients above 18 years, and the median weight was 50 kg (range 25–75 kg). There was an equal proportion of male and female patients. Five patients received 15 gms of L-glutamine twice daily and five patients received 10 gm of L-glutamine twice daily. Overall, nine patients received Hydroxyurea (HU) therapy at baseline and all patients received HU therapy during the follow-up visits (Table 1). The treating physicians reported 100% compliance for all patients under intervention.

**Table 1:** Baseline characteristics of patients with sickle cell disease (SCD).

Patients with SCD (*N* = 10)	
Age, years	10–37
Median	22
Range <18 years >18 years	4< 18 years, 6 >18 years
**Gender, *n***	
Female	5
Male	5
SCD* genotype	HbSS*
**Race**	
Black	6
Arab	4
**Weight, kg**	
Median	50
Range	25–75
**Age at time of SCD of diagnosis, *n***	
At birth	5
**Endari** ^**®**^ **dose, *g* (twice daily), *n***	
10 g	5
15 g	5
HU* therapy at baseline, *n*	
Yes	9
No	1
**HU therapy at all follow-up time points, *n***	
Yes	10

*HbSS, sickle hemoglobin; HU, hydroxyurea; SCD, sickle cell disease.

### Renal function in SCD

**Figure 1:**
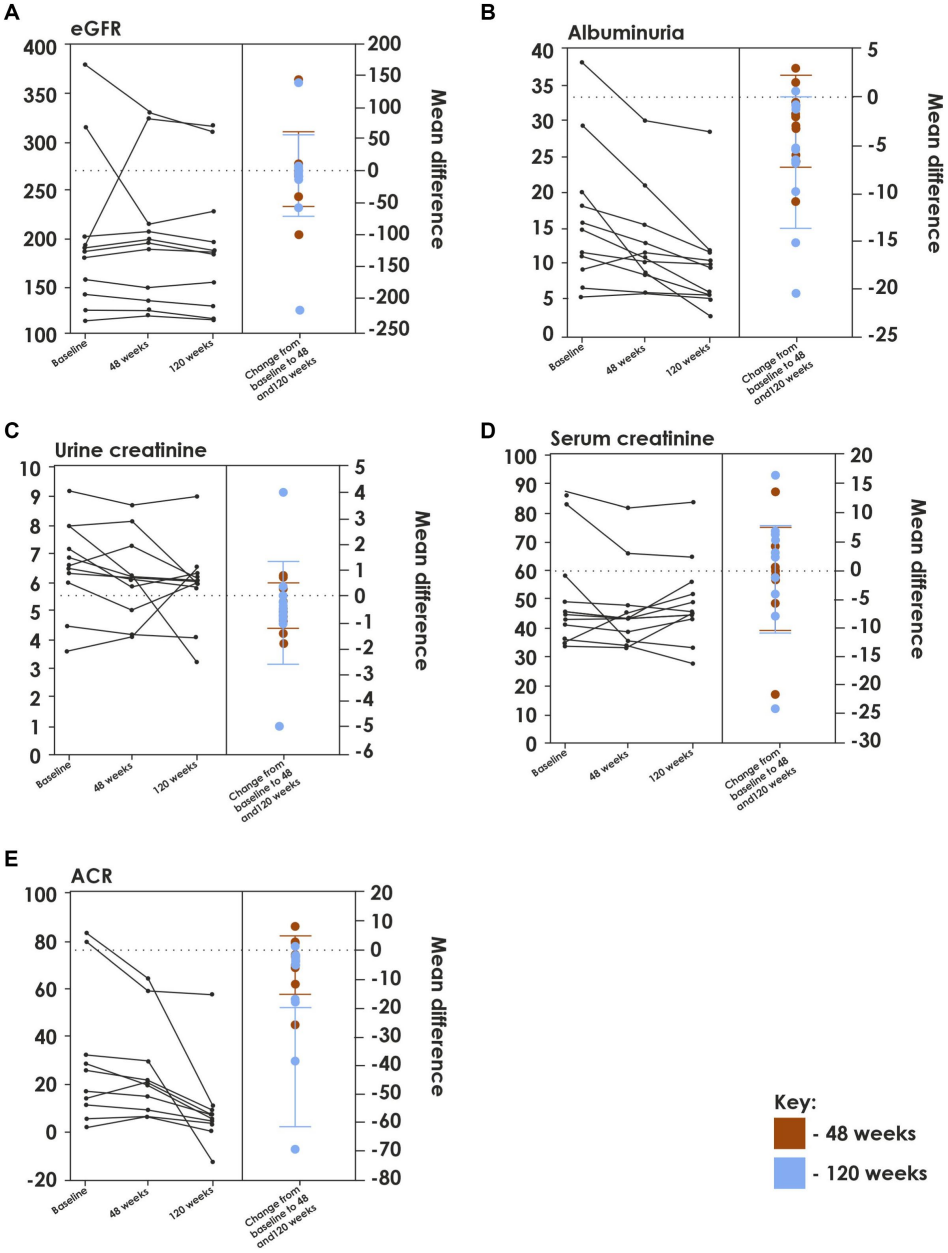
Effect of L-glutamine therapy on the level of different renal function markers from baseline to 48 and 120 weeks. **(A)** eGFR level reduced, though not statistically significant (*p*=0.7166) have clinical significance; **(B)** albuminuria significant decrease (*p*=0.0109); **(C)** urine creatinine (*p*=0.3934) and **(D)** serum creatinine (*p*=0.7529) reduced, though not statistically significant it is clinically relevant; **(E)** ACR (*p*=0.0981) showed a significant decrease.

Following L-glutamine therapy, significant improvements in renal parameters were observed from 48 weeks onwards, which was sustained through 120 weeks. Improvements were seen in the albumin creatinine ratio (ACR) from baseline to 48 weeks (mean [SD] ACR: −4.19 [9.81] mg/g) and 120 weeks (mean [SD] ACR: −12.31[21.09] mg/g). A significant improvement was observed in albuminuria levels at 48-and 120-week (*p* = 0.0441 and *p* = 0.0109, respectively). The eGFR levels also reduced from baseline to 48 weeks and 120 weeks (mean [SD] change from baseline to 48 weeks: −0.77 [59.12] mL/min/1.73m^2^; 120 weeks: −7.64 [64.49] mL/min/1.73m^2^), which is not statistically significant but has clinical significance (Table 2, Figure 1).

**Table 2:** Renal and hematological parameters in patients (*N* = 10) with sickle cell disease at baseline and follow-up time points.

Parameter	Baseline Mean (SD)	48 weeks Mean (SD)	Change from baseline to 48 weeks Mean (SD)	*p*-value* at 48 weeks	120 weeks Mean (SD)	Change from baseline to 120 weeks Mean (SD)	*p*-value* at 120 weeks
VOC, *n*	6.10 (4.91)	1.5 (1.37)	−5.20 (3.68)	0.0015	0 (0)	−6.10 (4.91)	0.0035
**Kidney profile**							
ACR	28.49 (26.39)	24.32 (18.23)	−4.19 (9.81)	0.2100	16.18 (15.08)	−12.31 (21.09)	0.0981
Albuminuria, mg/g	16.58 (9.93)	13.52 (7.43)	−3.06 (4.14)	0.0441	9.42 (6.66)	−7.16 (7.08)	0.0109
eGFR, mL/min/1.73m^2^	201.81 (82.55)	202.58 (76.06)	0.77 (59.12)	0.9680	194.17 (72.32)	−7.64 (64.49)	0.7166
Serum creatinine, U/L	46.40 (16.17)	44.80 (13.81)	−1.60 (8.87)	0.5824	47.60 (14.75)	1.20 (11.69)	0.7529
Urine creatinine, U/L	6.63 (1.68)	6.22 (1.52)	−0.41 (0.79)	0.1320	6.02 (1.62)	−0.62 (2.17)	0.3934
**Hematological profile**							
Hemoglobin, g/dL	8.73 (1.82)	9.45 (1.34)	0.72 (1)	0.0482	10.14 (1.53)	1.41 (0.79)	0.0003
WBC, ×10^9^ cells/L	12.26 (4.73)	10.58 (4.53)	−1.68 (3.34)	0.1465	7.91 (1.69)	−4.35 (4.83)	0.0191
LDH, U/L	539.20 (210.06)	280.20 (96.73)	−259 (154.93)	0.0005	194.40 (98.86)	−344.80 (274.63)	0.0033
Reticulocytes, ×10^9^ cells/L	249.30 (86.71)	209 (71.53)	−40.30 (101.58)	0.2413	191 (89.78)	−58.30 (128.38)	0.1848

ACR, albumin:creatinine ratio; eGFR, estimated glomerular filtration rate; LDH, lactate dehydrogenase; SD, standard deviation; VOC, vaso-occlusive crisis; WBC, white blood cells. *p < 0.05 was considered significant.

The improvements in renal parameters were consistent with the improvements in hematological parameters. Mean (SD) increase in hemoglobin concentrations from baseline to 48 weeks was 0.72 (1) g/dL and at 120 weeks was 1.41 (0.79) g/dL and the increase in hemoglobin level from baseline to all follow-up time points was statistically significant (*p* = 0.0482 at 48 weeks, *p* = 0.0003 at 120 weeks). Mean (SD) reticulocyte counts and LDH levels decreased from baseline to 48 weeks (mean [SD] change from baseline to 48 weeks, reticulocyte counts: −40.30 [101.58] × 10^9^ cells/L; LDH levels: −259 [154.93] U/L) and 120 weeks (mean [SD] change from baseline to 120 weeks, reticulocyte counts: −58.30 [128.38] × 10^9^ cells/L; LDH levels: −344.80 [274.63] U/L) (Table 2). This reduction of LDH at 48 weeks and 120 weeks was statistically significant (*p* = 0.0005 at 48 weeks, *p* = 0.0033 at 120 weeks).

AEs due to L-glutamine treatment were neither reported by healthcare practitioners nor by patients.

## Discussion

This real-world observational study on 10 patients with SCD, wherein nine patients received HU therapy at baseline and all patients received HU therapy at follow-up time points, demonstrated improvement in the renal and blood parameters at 48-and 120-week of L-glutamine therapy. L-glutamine was started despite the patients being on HU since they were still suffering from VOCs. L-glutamine was provided through an early access program (EAP) and to be eligible for the medication, it was mandatory that the patient’s disease was not controlled despite being on HU maximum tolerated dose (MTD). Thus, all patients on HU were on HU MTD. Moreover, studies have reported that a substantial number of patients who do not respond well to HU are responsive to L-glutamine (8, 9). In the present study, renal function markers such as ACR and albuminuria were found to decrease from baseline to 48 weeks and 120 weeks of L-glutamine therapy.

Acute kidney injury, renal papillary necrosis, impaired urine acidification, and reduced urinary concentrating capacity are the major renal complications in SCD (10). Albuminuria is a precursor to glomerulopathy, which is characterized by hyperfiltration and the condition worsens with age (11). It has been observed that albuminuria progresses over time in patients with SCD in case they do not receive precise treatment (12) and therapies for CKD that are usually considered for the management of albuminuria (13). A longitudinal multicenter study detected a correlation between persistent albuminuria and the risk of eGFR decline followed by CKD in adult patients with SCD (13). Moreover, the study reported that a baseline ACR threshold >100 mg/g was associated with persistent albuminuria (13). A study by Ranque et al. (14), reported that a high ACR leads to early kidney damage in children. Since renal impairment is a major independent risk factor for mortality in patients with SCD (10), the reduction of albuminuria and ACR is indicative of improvement of the renal condition and reduced risk of SCN. Although the reduction of ACR in our study is not statistically significant, it is clinically relevant. This could be attributed to the fact that while there was a significant reduction in urine albumin, creatinine level reduction was not statistically significant. However, it was observed that ACR reduction was more in 120 weeks than in 48 weeks indicating that use of L-glutamine can be continued beyond 120 weeks to find out whether ACR reduction becomes statistically significant with long-term use of L-glutamine.

In studies on adults and children, a strong association was seen between hemolysis parameters and increased albuminuria, suggestive of the fact that endothelial dysfunction due to hemolysis may be primarily responsible for SCN (12, 15). Thus, the present study findings suggest that long-term use of L-glutamine provided a beneficial effect in lowering the risk of renal damage in the patients as there is a significant reduction in albuminuria.

A statistically significant reduction in LDH level was also observed at 48 weeks (*p* = 0.0005) and 120 weeks (0.0033) follow-up with L-glutamine therapy. This suggested a decrease in hemolysis since LDH is routinely used as a hemolysis marker in SCD (16). The finding of the present study is in accordance with previous studies, which reported a significant improvement in clinical outcomes in terms of changes in the hemolysis parameters, a significant reduction in blood transfusions, and an increased level of hemoglobin with the use of oral L-glutamine in patients with SCD (5, 6). In SCD, red blood cells (RBC) are continually hemolysing, hence, the hemoglobin level is reduced leading to hemolytic anemia in some cases (16). Furthermore, elevated pulmonary artery pressure in patients with SCD is associated with low hemoglobin, high LDH, elevated systolic systemic blood pressure, and renal insufficiency (17). An increase in LDH level has a positive correlation with increased hemolysis and severity of pain in vaso-occlusive episodes due to an increased rate of red blood cell hemolysis (18). In our present study along with the decrease in LDH, there was a significant increase in hemoglobin level (*p* = 0.003) from baseline to 120 weeks of follow-up treatment indicating L-glutamine therapy is associated with significant improvement of renal and hematological parameters.

In line with a previous study (6), the reticulocyte count also showed a reduction following long-term treatment (120 weeks) with L-glutamine. Reticulocyte is also a hemolysis marker, reduction in the count is indicative of reduced hemolysis in the patients. Patients with SCD have higher immature reticulocytes, an excess of these when released into the circulation can lead to thrombosis. Both L-glutamine and HU can decrease the release of immature reticulocytes from the bone marrow; thus, reducing the number of reticulocytes, which is beneficial for patients with SCD (19). Although in our study, the reduction of reticulocytes is not statistically significant, the decrease in reticulocyte count is more in 120 weeks than in 48 weeks suggesting that further long-term therapy might yield a better reduction in the reticulocyte count with better outcome.

The exact mechanism of benefit of L-glutamine on kidney functioning in SCD is not known yet. The amino acid L-glutamine is responsible for the synthesis of nicotinamide adenine dinucleotide (NAD), arginine, and glutathione and is also known to protect RBC from oxidative damage (11). The antioxidant effects of L-glutamine are regarded as its main therapeutic mechanism in SCD (20). Reactive oxygen species (ROS) are known to increase RBC hemolysis and adhesion and play an independent role in SCD pathophysiology. Mammalian cells have different antioxidant pathways to counteract ROS, such as NAD(hydrogen (H)), Nicotinamide adenine dinucleotide phosphate (NADP(H)), and nitric oxide (NO) (20). L-glutamine has both pro-oxidant and antioxidant effects. The generation of renal arginine, a substrate for the enzyme nitric oxide synthase (NOS), which generates NO, is supported by enterally ingested glutamine. NO regulates regional blood flow and reduces thrombosis by vasodilation, inhibition of platelet aggregation, production of procoagulant proteins, and endothelial cell adhesion molecule expression (20). L-glutamine is known to replenish NO and reduce hemolysis (19). Furthermore, a pivotal phase 3 study showed that a lower incidence of VOC was observed in patients treated with L-glutamine (19). This is believed to be due to an increase in the proportion of the reduced form of NAD in the RBC of patients with SCD, leading to a reduction in oxidative stress (21).

The findings of the present study indicate a significant improvement in renal parameters with the use of oral L-glutamine for 120 weeks. These promising results might help increase the willingness of patients to adhere to L-glutamine therapy. The main limitation of the study is its small sample size. Also, since this is a retrospective real-world study the prior duration of HU therapy was not captured in the case records. Further studies with large sample size and more long-term use of L-glutamine are warranted to substantiate the efficacy of L-glutamine therapy in SCD.

## Conclusion

This is one of the first studies as per our knowledge that studied the long-term renal outcomes in SCD using L-glutamine. Improvements in clinical outcomes and hemolysis were observed. L-glutamine seems to improve the renal function of SCD patients from 48 weeks and this improvement was sustained through 120 weeks of treatment.

This real-world observational study will enroll more patients and will therefore use stronger statistical analysis to confirm these encouraging preliminary results.

## Data availability statement

The original contributions presented in the study are included in the article/supplementary material, further inquiries can be directed to the corresponding author.

## Ethics statement

The studies involving humans were approved by MRC- 04-20-1240 in Qatar and Commission Nationale Informatique et Libertés approval Number 3Yj157849 3 in French Guiana. The studies were conducted in accordance with the local legislation and institutional requirements. Written informed consent for participation in this study was provided by the participants’ legal guardians/next of kin.

## Author contributions

NE and MY: conceptualization, investigation, methodology, validation, and writing—review and editing. All authors contributed to the article and approved the submitted version.
